# Structural definition for a new modality of broad and potent antibody neutralization at the CD4-binding site on HIV-1 gp120

**DOI:** 10.1186/1742-4690-9-S2-P57

**Published:** 2012-09-13

**Authors:** T Zhou, S Moquin, R Lynch, X Wu, J Zhu, Y Yang, B Zhang, JR Mascola, PD Kwong

**Affiliations:** 1 National Institute of Allergy and Infectious Diseases/NIH, Bethesda, MD, USA

## Background

The initial site of CD4-attachment on HIV-1 gp120 is vulnerable to neutralizing antibodies, and a number of such antibodies have been found that target this site. One set of antibodies, represented by VRC01, mimic CD4 in their recognition and utilize a common V-gene origin (VH1-2*02). Another set of antibodies, represented by the recently identified VRC13, derives from VH1-69*01 and is able to neutralize over 90% of circulating HIV-1 isolates, including isolates resistant to VRC01. Do the VRC13-like antibodies also mimic CD4, or do they represent a new modality of effective CD4-binding-site neutralization?

## Methods

To define the mode of recognition used by VRC13, we crystallized its antigen-binding fragment in complex with HIV-1 gp120, from both VRC01-sensitive and VRC01-resistant strains, and determined these X-ray structures.

## Results

The structure of VRC13 indicates a mode of recognition rotated by 45 degrees and translated ~10 Å from that of VRC01, although both VRC01 and VRC13 utilize similar angles of approach. Unlike VRC01-like antibodies, which feature gp120 contacts primarily in the heavy chain 2nd complementarity determining region (CDR H2), VRC13 utilizes a long heavy chain CDR H3 to contact the CD4-binding site. Overall, the structural details of VRC13 do not mimic those of CD4.

## Conclusion

Broad and potent neutralization at the CD4-binding site is not limited to the VRC01-mode of CD4 mimicry. A new mode of effective HIV-1 neutralization, which is defined by the VRC13-gp120 structure and utilizes CDR H3 recognition, may serve as an additional template for the design of an effective HIV-1 vaccine. The natural diversity of the CDR H3 – a product of V-D-J recombination – may provide advantages in the elicitation of VRC13-like antibodies.

